# Regional brain amyloid-β accumulation associates with domain-specific cognitive performance in Parkinson disease without dementia

**DOI:** 10.1371/journal.pone.0177924

**Published:** 2017-05-25

**Authors:** Rizwan S. Akhtar, Sharon X. Xie, Yin J. Chen, Jacqueline Rick, Rachel G. Gross, Ilya M. Nasrallah, Vivianna M. Van Deerlin, John Q. Trojanowski, Alice S. Chen-Plotkin, Howard I. Hurtig, Andrew D. Siderowf, Jacob G. Dubroff, Daniel Weintraub

**Affiliations:** 1 Department of Neurology, Perelman School of Medicine, University of Pennsylvania, Philadelphia, Pennsylvania, United States of America; 2 Center for Neurodegenerative Disease Research and Institute on Aging, Perelman School of Medicine, University of Pennsylvania, Philadelphia, Pennsylvania, United States of America; 3 Department of Biostatistics and Epidemiology, Perelman School of Medicine, University of Pennsylvania, Philadelphia, Pennsylvania, United States of America; 4 Department of Radiology, Division of Nuclear Medicine and Clinical Molecular Imaging, Perelman School of Medicine, University of Pennsylvania, Philadelphia, Pennsylvania, United States of America; 5 Department of Pathology and Laboratory Medicine, Perelman School of Medicine, University of Pennsylvania, Philadelphia, Pennsylvania, United States of America; 6 AVID Radiopharmaceuticals, Philadelphia, Pennsylvania, United States of America; 7 Department of Psychiatry, Perelman School of Medicine, Perelman School of Medicine, University of Pennsylvania, Philadelphia, Pennsylvania, United States of America; 8 Parkinson’s Disease and Mental Health Research, Education, and Clinical Centers (PADRECC and MIRECC), Philadelphia Veterans Affairs Medical Center, Philadelphia, Pennsylvania, United States of America; Nathan S Kline Institute, UNITED STATES

## Abstract

Parkinson disease patients develop clinically significant cognitive impairment at variable times over their disease course, which is often preceded by milder deficits in memory, visuo-spatial, and executive domains. The significance of amyloid-β accumulation to these problems is unclear. We hypothesized that amyloid-β PET imaging by ^18^F-florbetapir, a radiotracer that detects fibrillar amyloid-β plaque deposits, would identify subjects with global cognitive impairment or poor performance in individual cognitive domains in non-demented Parkinson disease patients. We assessed 61 non-demented Parkinson disease patients with detailed cognitive assessments and ^18^F-florbetapir PET brain imaging. Scans were interpreted qualitatively (positive or negative) by two independent nuclear medicine physicians blinded to clinical data, and quantitatively by a novel volume-weighted method. The presence of mild cognitive impairment was determined through an expert consensus process using Level 1 criteria from the Movement Disorder Society. Nineteen participants (31.2%) were diagnosed with mild cognitive impairment and the remainder had normal cognition. Qualitative ^18^F-florbetapir PET imaging was positive in 15 participants (24.6%). Increasing age and presence of an *APOE* ε4 allele were associated with higher composite ^18^F-florbetapir binding. In multivariable models, an abnormal ^18^F-florbetapir scan by expert rating was not associated with a diagnosis of mild cognitive impairment. However, ^18^F-florbetapir retention values in the posterior cingulate gyrus inversely correlated with verbal memory performance. Retention values in the frontal cortex, precuneus, and anterior cingulate gyrus retention values inversely correlated with naming performance. Regional cortical amyloid-β amyloid, as measured by ^18^F-florbetapir PET, may be a biomarker of specific cognitive deficits in non-demented Parkinson disease patients.

## Introduction

Clinical Parkinson disease (PD) is a common and progressive neurodegenerative disease manifested by bradykinesia, rigidity, and rest tremor [1], and up to 80% of patients eventually develop dementia [2, 3]. The diagnostic neuropathological features in PD are pathological accumulation of α-synuclein and neuronal loss in the substantia nigra [4]. Deposits of other amyloidogenic proteins, particularly amyloid-β (Aβ) and tau, are also frequently found in PD brain, and these pathologies associate with dementia in PD [5, 6]. Comparatively less is known about their contribution to mild cognitive impairment (MCI) in PD.

PET radiotracers, such as ^18^F-florbetapir and ^11^C-PiB, can reveal fibrillar Aβ neuropathology in living patients [7]. Cortical radiotracer binding corresponds to fibrillar Aβ pathology seen at autopsy in patients with Alzheimer’s disease (AD) [8–10] and PD with dementia (PDD) [11]. This tracer can help to determine if patients with dementia have AD versus a non-Alzheimer dementia [10, 12], and Aβ amyloidosis is a prognostic biomarker in patients with mild cognitive impairment (MCI) for developing AD [13]. Prior studies focused on non-demented PD patients have used ^11^C-PiB PET brain imaging while we used ^18^F-florbetapir. We previously showed in a small number of PD patients without cognitive impairment that ^18^F-florbetapir did not correlate with global cognitive performance [14]. However, non-demented PD patients typically have impairment in specific and few neuro-cognitive domains [15], and the association of ^18^F-florbetapir with these individual functions is unclear.

In this study, we hypothesized that Aβ amyloid imaging by ^18^F-florbetapir PET would correlate with cognitive impairment in a well-characterized study population of PD patients. To test this hypothesis, we recruited PD patients without dementia for ^18^F-florbetapir PET scans and for detailed psychometric analyses. We also performed additional qualitative and quantitative imaging analyses aimed at understanding the technical performance characteristics of ^18^F-florbetapir PET as an *in vivo* imaging tool in PD patients. We determined the inter-rater reliability of expert interpretation of ^18^F-florbetapir PET imaging, which has not been previously determined in PD patients. Using ordered logistic and linear regression models, we examined the association of ^18^F-florbetapir with MCI and cognitive abilities when controlled for age, sex, education, disease duration, and the presence of an *APOE* ε4 allele. Finally, we computed regional ^18^F-florbetapir PET retention values to determine whether regional Aβ amyloidosis could either identify MCI or correlate with poor performance in individual neuro-cognitive domains.

## Methods

### Patients and clinical measures

We recruited non-demented patients from the Parkinson Disease and Movement Disorders Center of the University of Pennsylvania as a cohort of subjects within our Morris K Udall Center for Parkinson’s Disease Research (John Q. Trojanowski, P.I.). All sixty-one patients were diagnosed with PD according to the U.K. Brain Bank Criteria [16]. Disease duration was defined as time in years since PD symptom onset.

Each participant underwent a test battery comprised of assessments of global cognition and individual neuropsychological tests, as previously described [17]. An internal, blinded consensus conference, composed of 3–5 pairs of trained physician raters (movement disorders specialists and a psychiatrist with expertise in PD cognition), categorized each participant as having intact cognition (PD-NC) or MCI (PD-MCI). Consensus assessments were based on the available clinical and neuropsychological data from 11 tests annotated with Z-scores, in keeping with the Level 1 recommendations of the Movement Disorders Society (MDS) Task Force for MCI [18]. No motor characteristics or features, ^18^F-florbetapir imaging results, or other biomarker data were used in the consensus process. Between-pair inter-rater agreement for cognitive consensus was assessed in a separate cohort of 137 cases and was found to be high (kappa = 0.802, 95% confidence interval (C.I.) = 0.700–0.904). An independent physician rater adjudicated for cases with a between-pair discrepancy.

For measures of motor function, participants were assessed by the Unified Parkinson Disease Rating Scale (UPDRS part 3) and the modified Hoehn & Yahr (H&Y) scale. All testing was performed in the participant’s “ON” state. Participants were phenotyped as tremor-dominant (TD) or postural instability–gait dysfunction (PIGD) type by calculating a TD / PIGD ratio as previously described [19]. The numerator and denominator of the TD / PIGD ratio were defined as the mean motor subscores for tremor and postural instability. Because PIGD features have been previously associated with increased Aβ amyloid burden in PD patients [20], we grouped TD and indeterminate (IND) cases together to assess the contribution of PIGD phenotype to cognitive and imaging results.

We used the clinical evaluation closest in time to the ^18^F-florbetapir imaging for all analyses. All study protocols were approved by the University of Pennsylvania Institutional Review Board. Informed consent was obtained from each participant in the study prior to enrollment.

### Aβ PET imaging

Brain neuritic Aβ plaque burden was assessed by PET neuroimaging with ^18^F-florbetapir. Briefly, participants underwent a 10-minute PET scan 50 minutes following an intravenous bolus of 370 MBq (10 mCi) ^18^F-florbetapir. Scans were obtained between 2009 and 2014 at two clinical sites of the University of Pennsylvania on either a Siemens Biograph mCT PET/CT scanner or a Philips Ingenuity PET/CT scanner. Images were acquired with a 128X128 matrix (zoom 2) and reconstructed using iterative or row action maximization likelihood algorithms. Two board-certified nuclear medicine physicians independently interpreted the ^18^F-florbetapir PET images as positive or negative for Aβ amyloid positivity according to current guidelines and published methods [9]. Briefly, scans that evidenced increased binding of tracer in cortical grey matter, as shown by the apparent loss of contrast in grey or white matter in at least two cortical regions, or intense uptake in at least one cortical region, were classified as Aβ amyloid positive. Discrepant individual interpretations were adjudicated by consensus.

**Fig 1:**
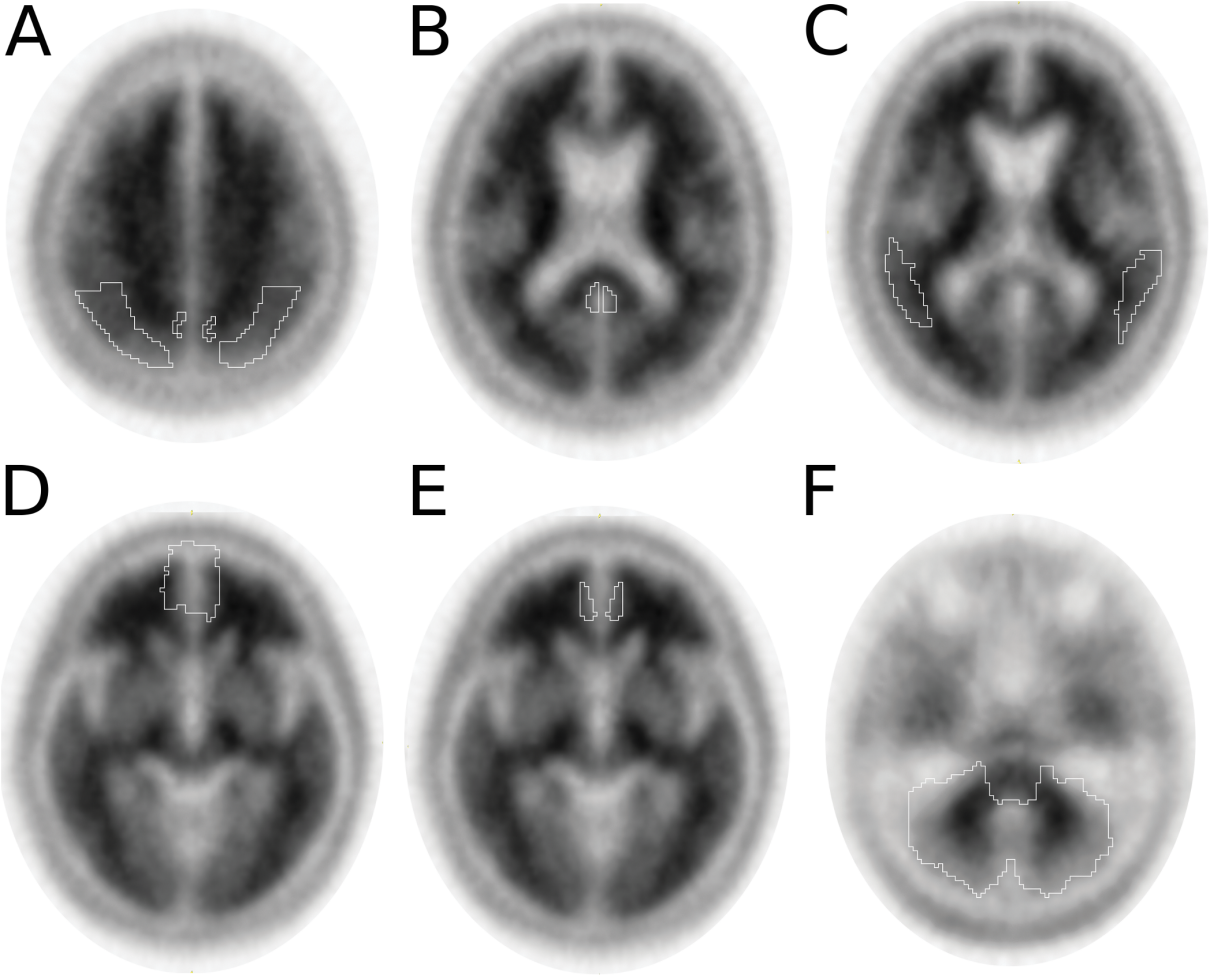
Analyzed regions of interest (ROIs) in the ^18^F-florbetepir PET scans. Six individual cortical ROIs and a cerebellum ROI were analyzed for regional ^18^F-florbetapir binding and to calculate a composite ROI (see Methods). Representative trans-axial images from a Aβ negative (left) and positive (right) study demonstrate each ROI: (A) parietal cortex and precuneus; (B) frontal cortex; (C) anterior and posterior cingulate gyrus; (D) temporal cortex; and (E) cerebellum. Darker regions indicate higher ^18^F-florbetapir retention.

Because cerebral Aβ amyloid likely accumulates gradually over time, we analyzed quantitative ^18^F-florbetapir binding in parallel with the qualitative interpretation by our expert readers. Cerebral Aβ amyloid was quantified using a semi-automated PET atlas method that was previously reported and validated [21, 22], using the ^18^F-florbetapir specific PET template from those previous studies. The ^18^F-florbetapir specific PET template was provided to us by Molecular NeuroImaging, LLC. Probabilistic template segmentation was used since structural brain MRI was not available for all subjects within 6 months of ^18^F-florbetapir PET. Each patient’s ^18^F-florbetapir PET was spatially normalized to the ^18^F-florbetapir PET template space, in order to calculate the inverse transformation matrix for that subject’s PET. The inverse transformation matrix of each subject was applied to the template regions-of-interest (ROIs) and overlaid on that respective patient’s PET scan, in order to quantify cortical ^18^F-florbetapir retention in each patient’s original PET space. The spatial normalization, transformation, and quantification were performed in PMOD (version 3.6, PMOD Technologies Ltd.). Cortical ^18^F-florbetapir PET retention was expressed as a standardized uptake value ratio (SUVr) using the cerebellum as a reference region. Because specific brain regions important for cognitive decline in PD have not been determined, we calculated regional SUVrs for six volume-weighted cortical ROIs known to be important for memory function in AD (anterior cingulate, posterior cingulate, precuneus, temporal cortex, frontal cortex, and parietal cortex) [22, 23]. Right and left hemisphere values were averaged for each region. A composite ROI SUVr was also calculated as the weighted average SUVr of the six ROIs, with the contribution of each ROI based on the volume of that ROI relative to the overall summed volume of the six ROIs. Representative ^18^F-florbetapir PET axial images from a positive scan depicting each ROI are shown in Fig 1.

To confirm that qualitative PET interpretation agrees with quantitative imaging in our cohort [14, 24], we compared qualitative, expert radiologist interpretation of ^18^F-florbetapir PET imaging to quantitative composite ROI SUVr values using a previously published SUVr cutoff value of 1.10 to distinguish positive Aβ PET scans from negative scans [21, 25, 26]. Cohen’s kappa was used to assess the agreement of presence of Aβ amyloid deposition between the two expert raters and between the consensus expert read and scans defined as positive by virtue of the composite ROI SUVr exceeding the cutoff value.

### *APOE* genotyping

The *APOE* ε4 allele is associated with cerebral Aβ amyloid deposition in older adults and patients with either MCI or AD [27]. Genomic DNA was isolated from peripheral blood samples using manufacturer’s protocols (FlexiGene, QIAGEN, Valencia, CA or QuickGene, Autogen, Holliston, MA). Genotyping for the *APOE* ε4 allele was performed using real-time allelic discrimination PCR using TaqMan reagents and ABI assays C_904973_10 (for single nucleotide polymorphism rs7412) and C_3084793_20 (for rs429358) on an ABI 7500 real-time PCR instrument (Applied Biosystems, Foster City, CA) using standard conditions.

### Statistical analyses

Comparisons across groups for cross-sectional analyses of demographics, clinical characteristics, and biomarker data were performed using two-sample t-test (for normally distributed variables), Wilcoxon-Mann-Whitney test (for non-normally distributed or ordered categorical variables), Fisher’s exact test, or one-way analysis of variance (ANOVA) and Tukey’s post-hoc tests where appropriate. Correlation analyses of cognitive performance and ^18^F-florbetapir imaging results were performed using linear regression and Pearson partial correlation to adjust for age, sex, education, and presence of an *APOE* ε4 allele. Binary logistic regression was used to evaluate the contribution of PET imaging measures to cognitive consensus diagnosis. All statistical analyses were performed using Stata/IC (13.1, College Station, TX). Figures were prepared with GraphPad Prism (6.0, La Jolla, CA). All statistical tests were two-sided. For comparisons involving individual brain regions or individual neuropsychological tests, we adjusted for multiple testing using Bonferroni correction [28]. Continuous variables are presented as mean (95% C.I.) unless otherwise specified. Statistical significance was set at <0.05 level. Participant level data is available in the supplementary materials (S1 File).

## Results

### Study demographics and clinical correlations between motor and cognitive performance

72.1% of the participants were male. Mean (95% C.I.) age of disease onset was 60.0 (57.7 to 62.4) years, age at scan time was 67.0 (64.9 to 69.0) years, disease duration was 7.7 (6.5 to 8.9) years, and years of education was 16.3 (15.7 to 16.9) years. 32.8% of participants had at least one *APOE* ε4 allele. 41.8% of cases experienced motor fluctuations. 59.3% of cases were of the PIGD motor phenotype. On average, cognitive and motor data were collected 133 days prior to ^18^F-florbetapir scan (range 0 days to 664 days before the scan). Only three cases were clinically evaluated more than one year prior to their scan.

Nineteen cases (31.2%) were found to have mild cognitive impairment (PD-MCI) and the remaining 42 (68.9%) had normal cognition (PD-NC). Both PD-NC and PD-MCI had similar proportions of males (68.4% and 73.8%, respectively) (Fisher’s exact = 0.76). PD-NC had more years of education (16.6, 15.9 to 17.2) than PD-MCI (15.7, 14.3 to 17.1) that was not significantly different (t(59) = 1.36, p = 0.18). An *APOE* ε4 allele was present in similar proportions in PD-NC (31.0%) and PD-MCI (36.8%) (Fisher’s exact = 0.77).

**Fig 2:**
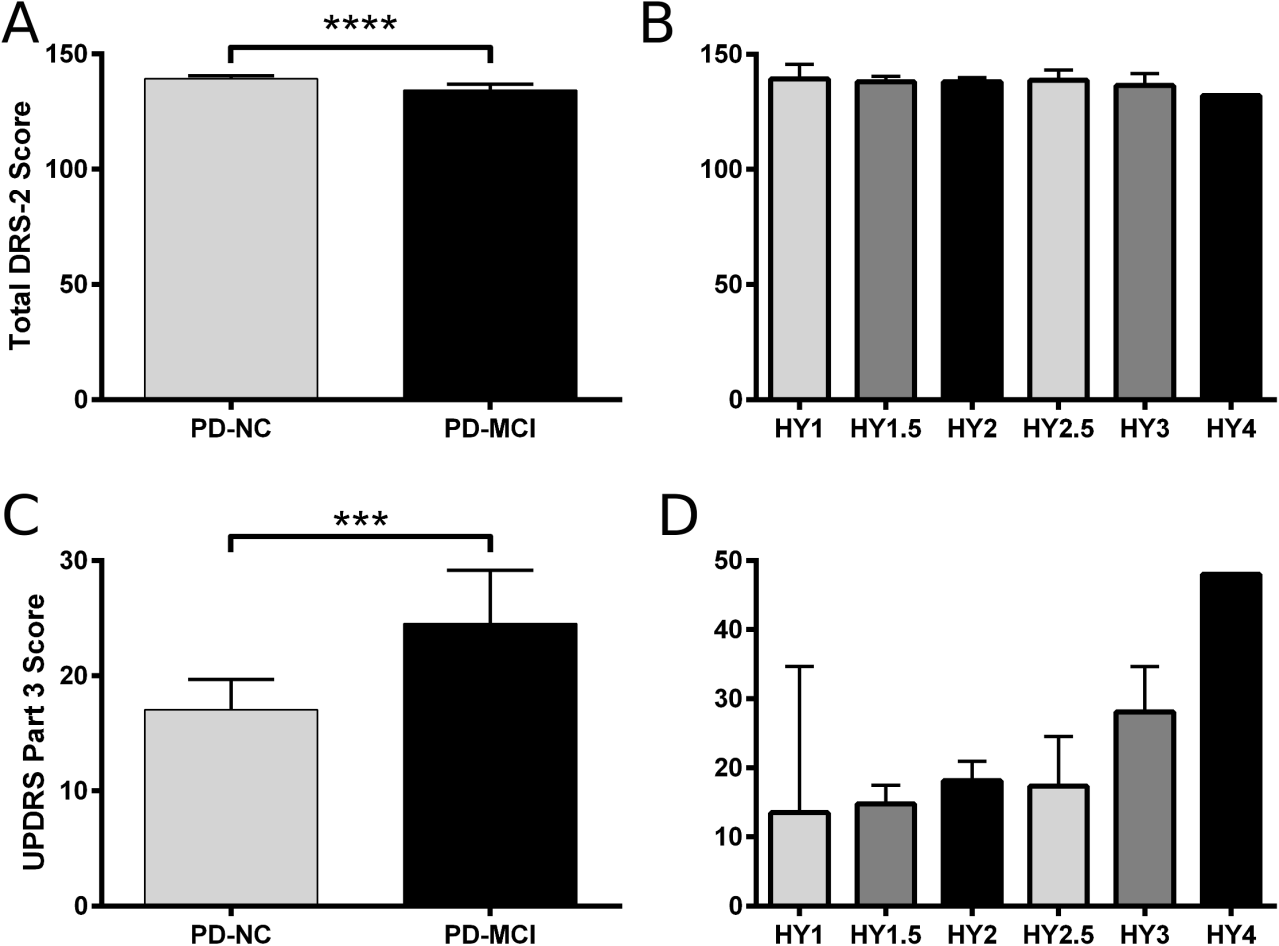
Cognitive and motor characteristics of the study population. A. Total DRS-2 score was significantly lower in PD-MCI participants (134.3, 131.7 to 136.9) as compared to PD-NC (139.3, 138.1 to 140.6) (t(57) = 4.12, p < 0.001). B. Total DRS-2 score was not significantly different among H&Y stages (F(5,53) = 0.54, p = 0.75). C. UPDRS part 3 score was significantly higher in PD-MCI (24.5, 19.9 to 29.2) as compared to PD-NC (17.1, 14.5 to 19.7) (t(59) = 3.07, p = 0.003). D. UPDRS part 3 score was significantly higher with advanced H&Y stage (1.0: 13.5, 0.2 to 26.8; 1.5: 14.8, 12.4 to 17.1; 2.0: 18.2, 15.5 to 20.9; 2.5: 17.3, 11.8 to 22.9; 3.0: 28.1, 22.3 to 33.9; 4.0: 48) (F(5,55) = 6.57, p < 0.001). There was no association between motor phenotype (TD vs. PIGD) and H&Y stage (data not shown). Data depicted are mean ± 95% C.I. Significance identified by one-way ANOVA and Tukey post-hoc test. *** p < 0.005, **** p < 0.001.

Associations between key cognitive and motor features are presented in Fig 2. There was no significant association between cognitive rating (PD-NC vs. PD-MCI) and H&Y stage (Fisher’s exact = 0.16). Cognitive rating did not associate with the presence of motor fluctuations (Fisher’s exact = 1.00). We did not find an association between cognitive rating and the PIGD motor phenotype (Fisher’s exact = 0.084). Although motor disease duration was shorter in PD-MCI (7.1, 5.3 to 9.0) as compared to PD-NC (8.0, 6.4 to 9.6), this difference was not statistically significant (t(59) = 0.66, p = 0.51). Longer disease duration was significantly associated with more severe H&Y stage (F(5,55) = 3.90, p = 0.004). There were no significant differences in age of onset or age at scan time between PD-NC and PD-MCI (data not shown). There were no significant differences in age of onset, age at scan time, sex, years of education, or presence of an *APOE* ε4 allele among all H&Y stages (data not shown).

### Qualitative and quantitative metrics for ^18^F-florbetapir PET imaging

Fifteen (24.6%) ^18^F-florbetapir scans were rated as positive by both expert readers. Cohen’s kappa for the initial qualitative read was 0.92 (59 / 61 scans), and both initially discordant scans were adjudicated as negative. There was no significant contribution of the clinical site of PET imaging to the expert rating (Fisher’s exact = 0.54). Positive scans had a significantly higher composite cortical:cerebellar ROI SUVr (1.268, 1.174 to 1.362) compared with negative scans (1.050, 1.030 to 1.069) (t(59) = 7.29, p < 0.001). For the entire cohort, average composite SUVr values were similar at either clinical site (Pennsylvania Hospital: 1.116, 1.055 to 1.177 and Hospital of the University of Pennsylvania: 1.097, 1.053 to 1.142) (t(59) = 0.49, p = 0.63). Using a SUVr cutoff of 1.10 [21, 25, 26], there was moderate agreement between qualitative expert rating and composite ROI SUVr-classified scans (kappa = 0.58, p < 0.001).

Age at disease onset and at scan time were not significantly different in those with positive scans as compared to negative scans (Table 1). However, participants whose scans had a composite ROI SUVr ≥ 1.10 were significantly older at disease onset (t(59) = 3.35, p = 0.001) and at time of scan (t(59) = 3.31, p = 0.002). The presence of an *APOE* ε4 allele was more common in positive scans than negative scans, but this difference was not statistically significant (for expert rating, Fisher’s exact = 1.00; for SUVr cutoff, Fisher’s exact = 0.22). There were no significant differences in sex, years of education, disease duration, or PIGD motor phenotype in participants with positive scans, determined either by qualitative read or by quantitative SUVr threshold. Participants with composite SUVr ≥ 1.10 had significantly higher UPDRS part 3 scores (t(59) = 2.14, p = 0.04), but this difference was not seen when scans were categorized by expert read (Table 1). Because participants with positive scans by SUVr cutoff were older at age of onset and at scan time, we asked whether these ages contributed to UPDRS part 3 score. We found no correlation between either age of onset or age at scan time and UPDRS part 3 score using univariate linear regression (F(1,59) = 0.25, p = 0.62 and F(1,59) = 1.29, p = 0.26). Using two-way ANOVA, UPDRS part 3 score remained significantly higher in scans positive by SUVr cutoff when age of onset ((F(1,58) = 6.69, p = 0.01) but not age at scan time (F(1,58) = 3.28, p = 0.08) was included as co-variates, likely owing to the strong effect of age at scan time in univariate analysis (p = 0.002, Table 1).

**Table 1:** Demographic characteristics in ^18^F-florbetapir positive versus negative scans.

^18^F-florbetapir assessment method	Expert visual rating			Composite ROI SUVr		
Scan result (N)	Negative (46)	Positive (15)	Statistic	< 1.10 (44)	≥ 1.10 (17)	Statistic
Age of onset, y	59.2	62.6	0.20	57.8	65.8	**0.001**
	(56.7 to 61.6)	(56.5 to 68.8)		(55.5 to 60.1)	(60.6 to 71.0)	
Age at scan time, y	66.2	69.1	0.24	65.0	72.1	**0.002**
	(64.2 to 68.3)	(63.0 to 75.3)		(62.9 to 67.0)	(67.3 to 76.9)	
Sex, male / female	31 / 15	13 / 2	0.20	32 / 12	12 / 5	1.00
*APOE* ε4, % present	32.6%	33.3%	1.00	27.3%	47.1%	0.22
Education, y	16.6	15.3	0.07	16.3	16.2	0.90
	(16.0 to 17.3)	(13.9 to 16.8)		(15.6 to 17.1)	(15.1 to 17.3)	
Disease duration, y	7.8	7.5	0.87	7.9	7.2	0.63
	(6.3 to 9.3)	(5.6 to 9.5)		(6.4 to 9.4)	(5.3 to 9.2)	
PIGD phenotype, % present	59.1%	60.0%	1.00	60.5%	56.3%	0.78
UPDRS part 3 score	18.5	22.1	0.20	17.8	23.4	**0.04**
	(15.8 to 21.2)	(16.8 to 27.5)		(15.0 to 20.7)	(19.0 to 27.9)	

Significant differences by two-sample T-test or Fisher’s exact test indicated in bold prior to correction for multiple comparisons. All data are mean (95% C.I.) unless otherwise specified.

On univariate linear regression, increasing age at scan time was significantly correlated with higher composite ROI SUVr value (F(1,59) = 9.41, p = 0.003). In contrast, disease duration and years of education did not correlate with higher composite SUVr values (F(1,59) = 0.79, p = 0.38 and F(1,59) = 0.11, p = 0.74). Males did not have significantly different composite SUVr values than females (t(59) = 0.876, p = 0.39). Participants with an *APOE* ε4 allele had slightly higher composite SUVr values (1.145, 1.061 to 1.229) as compared to those without an ε4 allele (1.083, 1.049 to 1.117) but did not reach statistical significance (t(59) = 1.67, p = 0.10). Age remained significantly associated with composite ROI SUVr when sex, disease duration, years of education, and presence of an *APOE* ε4 allele were added as covariates (F(5,55) = 3.23, p = 0.002). Controlling for age, the presence of an *APOE* ε4 allele was associated with higher composite ROI SUVr (F(2,58) = 7.00, p = 0.047). UPDRS part 3 score did not correlate with composite SUVr value (F(1,59) = 1.03, p = 0.31).

### Association between qualitative Aβ amyloid scan results and cognition

MCI status was not significantly associated with a positive ^18^F-florbetapir PET scan result based on either expert rating (Fisher’s exact = 0.20) or composite ROI SUVr (Fisher’s exact = 0.13) (Table 2). Total DRS-2 score was lower in subjects with positive scans (136.5, 133.0 to 140.1) as compared to negative scans (138.2, 136.9 to 139.5), but this difference was not statistically significant (t(57) = 1.16, p = 0.25). Similarly, total DRS-2 score was lower, but not significantly so, in scans with composite SUVr ≥ 1.10 (136.6, 133.1 to 140.1) as compared to scans with composite SUVr < 1.10 (138.2, 137.0 to 139.5) (t(57) = 1.12, p = 0.27). There was no difference in DRS-2 subscores of attention, initiation / perseveration, conceptualization, construction, or memory in participants with positive scans vs. negative scans by either categorization method (Table 2).

**Table 2:** Cognitive diagnosis and test results in ^18^F-florbetapir positive versus negative scans.

^18^F-florbetapir assessment method	Expert visual rating			Composite ROI SUVr		
Scan result (N)	Negative (46)	Positive (15)	Statistic	< 1.10 (44)	≥ 1.10 (17)	Statistic
Cognitive status, n. (%)						
PD-NC	34 (74%)	8 (53%)	0.20	33 (75%)	9 (53%)	0.13
PD-MCI	12 (26%)	7 (47%)		11 (25%)	8 (47%)	
Total DRS-2 score	138.2	136.5	0.25	138.2	136.6	0.27
	(136.9 to 139.5)	(133.0 to 140.1)		(137.0 to 139.5)	(13.1 to 140.1)	
DRS-2 sub-score						
Attention	35.8	35.5	0.52	35.7	35.7	0.98
	(35.3 to 36.3	(34.7 to 36.3)		(35.2 to 36.2)	(34.9 to 36.5)	
Initiation / Perseveration	36.1	35.4	0.24	36.1	35.5	0.32
	(35.6 to 36.7)	(33.9 to 36.9)		(35.7 to 36.6)	(33.8 to 37.2)	
Construction	5.8	5.9	0.73	5.8	5.8	0.86
	(5.7 to 6.0)	(5.7 to 6.1)		(5.7 to 6.0)	(5.6 to 6.0)	
Conceptualization	37.3	37.2	0.76	37.4	37.2	0.73
	(36.9 to 37.8)	(36.1 to 38.3)		(36.9 to 37.8)	(36.2 to 38.1)	
Memory	23.2	22.6	0.36	23.2	22.4	0.18
	(22.6 to 23.7)	(21.2 to 24.0)		(22.7 to 23.8)	(21.0 to 23.9)	
Boston Naming Test score	57.3	54.4	**0.01**	57.0	55.3	0.13
	(56.4 to 58.2)	(51.5 to 57.3)		(55.9 to 58.1)	(52.9 to 57.7)	
Judgment of Line Orientation score	24.0	23.1	0.55	24.5	21.9	0.07
	(22.5 to 25.9)	(20.4 to 25.7)		(23.0 to 25.9)	(19.1 to 24.6)	
Trails A time	49.0	44.8	0.65	44.7	56.7	0.18
	(39.1 to 58.9)	(32.3 to 57.3)		(36.2 to 53.2)	(37.4 to 76.0)	
Trails B time	120.0	112.7	0.75	113.9	130.9	0.45
	(96.1 to 143.8)	(78.0 to 147.4)		(91.0 to 136.8)	(89.4 to 172.5)	
Symbol–Digit Modalities Test score	35.9	34.4	0.68	36.9	31.7	0.15
	(32.1 to 39.7)	(27.8 to 41.0)		(33.1 to 40.7)	(25.4 to 38.1)	
Hopkins Verbal Learning Test-R						
Immediate free recall score	20.0	20.3	0.84	20.3	19.6	0.63
	(18.6 to 21.5)	(16.8 to 23.9)		(18.9 to 21.7)	(16.1 to 23.1)	
Delayed free recall score	5.7	6.3	0.54	5.6	6.4	0.44
	(4.7 to 6.7)	(4.1 to 8.6)		(4.5 to 6.7)	(4.5 to 8.3)	
Letter-Number Sequencing Test score	9.9	9.5	0.65	10.0	9.3	0.39
	(9.1 to 10.8)	(8.1 to 11.0)		(9.2 to 10.8)	(7.9 to 10.8)	
Verbal fluency						
F-A-S score	45.5	40.9	0.20	45.2	42.1	0.36
	(41.9 to 49.1)	(34.9 to 47.0)		(41.5 to 49.0)	(36.7 to 47.5)	
Semantic score	19.3	18.7	0.75	19.6	17.8	0.29
	(17.6 to 20.9)	(15.0 to 22.5)		(17.8 to 21.4)	(15.1 to 20.5)	

Significant differences by two-sample T-test or Fisher’s exact test indicated in bold prior to correction for multiple comparisons. All data are mean (95% C.I.) unless otherwise specified. Abbreviations: DRS-2 = Dementia Rating Scale– 2

On a detailed neuropsychological test battery (summarized in Table 2), participants with positive scans by expert rating scored significantly worse on the Boston Naming Test (BNT) (t(57) = 2.64, p = 0.01). However, this difference was no longer significant when corrected for multiple comparisons. There were no other significant differences in cognitive test performance between participants with positive scans vs. negative scans, irrespective of the categorization method used (Table 2).

We tested the association between MCI status and the ^18^F-florbetapir scan result in a multivariable logistic regression model that included age, sex, education, disease duration, and presence of *APOE* ε4 allele as covariates. Using either categorization method, the ^18^F-florbetapir scan result did not associate with a diagnosis of MCI (Table 3).

**Table 3:** Association between mild cognitive impairment and ^18^F-florbetapir PET imaging.

**Model 1**	**Outcome: Mild Cognitive Impairment**			
*Variable*	**OR**	**95% C.I.**	**Z-score**	**p-value**
^18^F-florbetapir expert rating	1.94	0.51 to 7.52	0.97	0.33
Age at scan time	1.05	0.97 to 1.12	1.22	0.22
**Model 2**	**Outcome: Mild Cognitive Impairment**			
^18^F-florbetapir SUVr cutoff	1.98	0.50 to 7.84	0.98	0.33
Age at scan time	1.03	0.96 to 1.11	0.85	0.39

Binary logistic regression models with sex, education, disease duration, and presence of *APOE* ε4 allele as additional covariates. Abbreviations: OR = odds ratio; CI = confidence interval.

### Association between quantitative composite and regional Aβ amyloid PET retention values and cognition

Composite ROI SUVr values did not significantly differ between the PD-NC and PD-MCI groups (Table 4). ^18^F-florbetapir binding was significantly higher in the posterior cingulate gyrus of PD-MCI participants as compared to PD-NC participants (t(59) = 2.28, p = 0.03), although this difference was no longer significant after correction for multiple comparisons. Regional SUVr values were higher in all remaining ROIs of PD-MCI participants but were not significantly different from PD-NC participants.

**Table 4:** Composite and regional Aβ amyloid retention in participants with and without cognitive impairment.

Cognitive diagnosis (N)	PD-NC (42)	PD-MCI (19)	T	p-value
Composite ROI SUVr	1.085	1.145	1.60	0.12
	(1.046 to 1.124)	(1.069 to 1.220)		
Regional SUVr				
Frontal	0.891	0.941	1.17	0.25
	(0.849 to 0.933)	(0.849 to 1.032)		
Temporal	1.151	1.205	1.47	0.15
	(1.113 to 1.189)	(1.131 to 1.278)		
Precuneus	1.111	1.211	1.77	0.08
	(1.051 to 1.170)	(1.098 to 1.323)		
Parietal	1.082	1.142	1.56	0.12
	(1.039 to 1.124)	(1.070 to 1.214)		
Anterior cingulate	1.033	1.126	1.52	0.13
	(0.973 to 1.094)	(0.995 to 1.257)		
Posterior cingulate	0.949	1.072	2.28	**0.03**
	(0.896 to 1.003)	(0.958 to 1.185)		

All data are mean (95% C.I.). Statistical significant differences by two-sample t test indicated in bold prior to correction for multiple comparisons. Abbreviations: PD-NC = normal cognition; PD-MCI = mild cognitive impairment; SUVr = standardized uptake value ratio.

**Fig 3:**
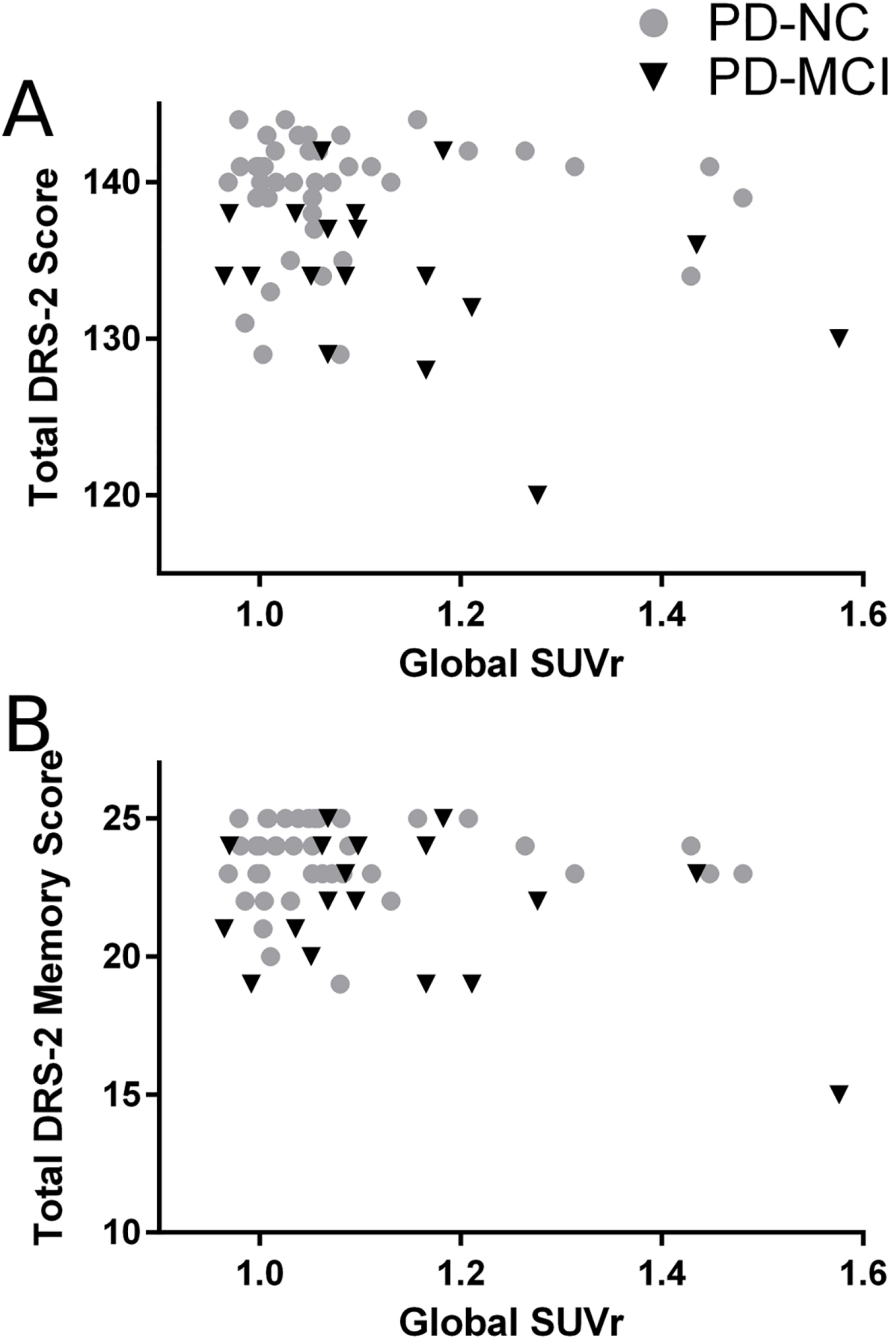
No correlation between composite ^18^F-florbetapir ROI SUVr and global cognition or memory performance. (A) Total DRS-2 score did not significantly correlate with composite ROI SUVr values, either analyzed in aggregate (F(1,57) = 2.39, p = 0.13) or by cognitive diagnosis (for PD-NC, F(1,39) = 0.02, p = 0.90; for PD-MCI, F(1,16) = 2.15, p = 0.16). (B) DRS-2 memory subscore did not significantly correlate with composite ROI SUVr values, either analyzed in aggregate (F(1,57) = 3.80, p = 0.06) or by cognitive diagnosis (for PD-NC, F(1,39) = 0.00, p = 0.97; for PD-MCI, F(1,16) = 3.03, p = 0.10).

We found no significant correlation between global cognition as measured by total DRS-2 score and composite ^18^F-florbetapir ROI SUVr (Fig 3A). Because age at scan time and presence of an *APOE* ε4 allele influenced composite SUVr values, we used Pearson partial correlation to correct for these variables and for sex, disease duration, and education. After adjustment, there was still no linear correlation between composite ROI SUVr and total DRS-2 score (p = 0.52). We next determined whether any DRS-2 subscores correlated with composite SUVr; only the memory subscore approached a statistically significant correlation with composite SUVr (Fig 3B). After Pearson partial adjustment, there remained no significant correlation between composite SUVr and DRS-2 memory subscore (p = 0.57). There was also no significant un-adjusted correlation between composite SUVr and DRS-2 subscores for attention (p = 0.42), initiation (p = 0.43), construction (p = 0.94), or conceptualization (p = 0.66).

There was a significant association between total DRS-2 score and regional SUVr values in the frontal cortex, anterior cingulate gyrus, and posterior cingulate gyrus (F(1,57 = 4.24, p = 0.04; F(1,57) = 5.11, p = 0.03, and F(1,57) = 4.89, p = 0.03). However, once adjusted for age, sex, disease duration, education, and presence of an *APOE* ε4 allele, these relationships were no longer significant (p = 0.22, p = 0.15, and p = 0.14). There was no significant association before or after adjustment between total DRS-2 score and the other regional SUVr values (data not shown).

**Fig 4:**
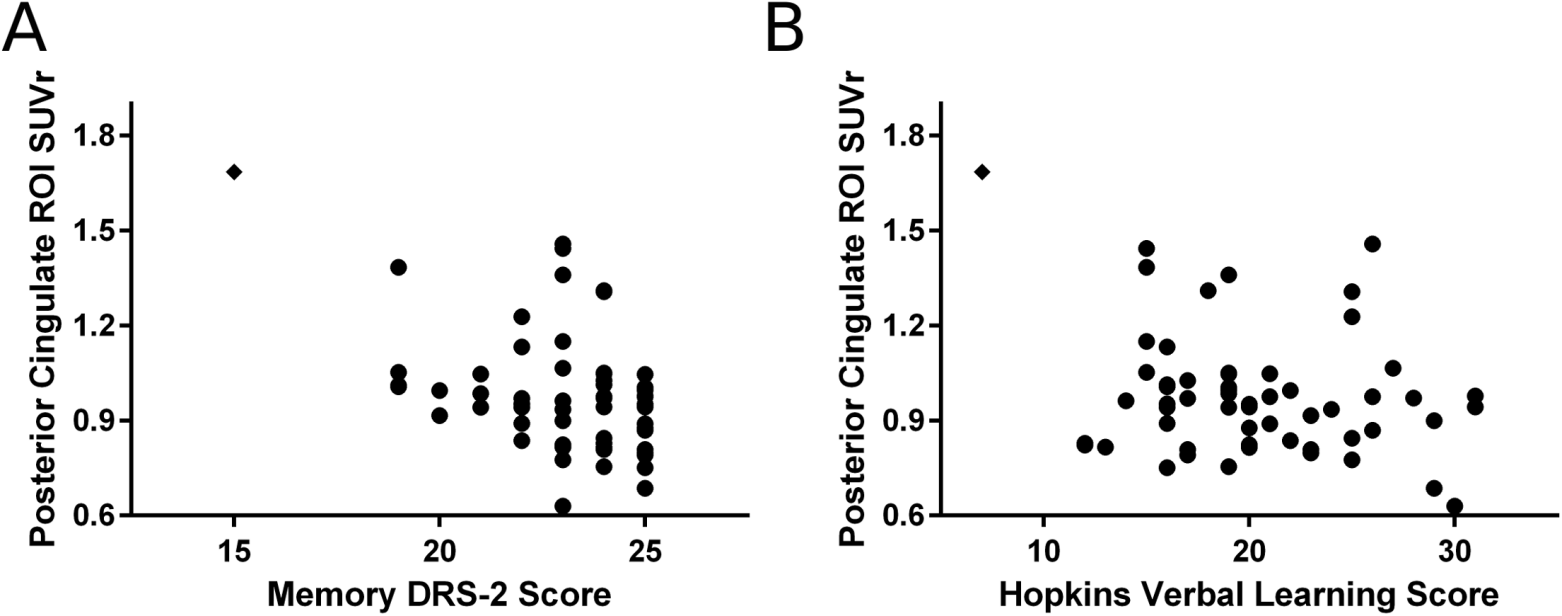
Association between posterior cingulate gyrus ^18^F-florbetapir SUVr and memory performance. Regional ^18^F-florbetapir SUVr in the posterior cingulate gyrus significantly correlated with memory performance on the DRS-2 (A, rho = -0.446, p < 0.0005), which remained significant after adjusting for age, sex, disease duration, education, and presence of an *APOE* ε4 allele (rho = -0.316, p = 0.02). SUVr in this region also significantly correlated with immediate free total recall on the Hopkins Verbal Learning Test (HVLT-R) on unadjusted analysis (B, rho = -0.263, p = 0.04) but not after adjustment (rho = -0.263, p = 0.054). Outlying participant indicated by closed diamond.

We examined DRS-2 subscores and regional SUVr values by pairwise correlation and found that DRS-2 memory subscore was significantly correlated with regional SUVr values in the frontal cortex, precuneus, anterior cingulate gyrus, and posterior cingulate gyrus (p = 0.02, p = 0.04, p = 0.01, and p < 0.005). One participant had a relatively low DRS-2 memory subscore (Fig 4A, black diamond). When this participant was excluded, only the posterior cingulate gyrus remained significantly correlated with DRS-2 memory subscore (rho = -0.270, p = 0.04). In addition, the posterior cingulate gyrus regional SUVr remained significantly correlated with DRS-2 memory score after adjustment for age, sex, disease duration, education, and presence of *APOE* ε4 allele (Fig 4A). There was no significant association between DRS-2 subscores in attention, initiation / perseveration, construction, or conceptualization and any region (data not shown).

To determine if other memory tasks also associated with regional ^18^F-florbetapir SUVr values, we examined performance on the Hopkins Verbal Learning Test (HVLT-R; immediate and delayed free recall) for correlations with regional SUVr values. As with DRS-2 memory, regional SUVr in the posterior cingulate gyrus significantly correlated with HVLT-R immediate recall score (Fig 4B). However, this correlation did not remain statistically significant after adjustment for co-variates above (rho = -0.263, p = 0.054) or when a single outlying participant was excluded (rho = -0.127, p = 0.34). There was no unadjusted association between HVLT-R delayed recall and regional SUVr values in the posterior cingulate gyrus (p = 0.42).

Our previous analysis suggested that confrontational naming, as measured by the BNT, is more impaired in participants with positive ^18^F-florbetapir scans. We investigated which cortical regions could contribute to this effect. Low total BNT score was significantly correlated with higher SUVr values in all six regions, but after adjustment for age, sex, disease duration, education and presence of an *APOE* ε4 allele, only correlations in the frontal cortex, precuneus, and anterior cingulate gyrus remained significant (rho = -0.295, p = 0.03; rho = -0.306, p = 0.03; and rho = -0.370, p = 0.006).

## Discussion

In this study of non-demented PD patients, composite Aβ amyloidosis as measured by ^18^F-florbetapir PET did not correlate with MCI diagnosis or global cognitive test performance. Both older age and the presence of an *APOE* ε4 allele were associated with significantly higher ^18^F-florbetapir PET uptake values, similar to what is seen in AD patients [27]. We found that regional Aβ amyloidosis in the posterior cingulate gyrus identified individuals with MCI, albeit prior to correction for multiple comparisons, and correlated with memory performance on neuropsychological testing. Importantly, the latter relationship persisted for some but not all methods of memory assessment after correction for age, sex, disease duration, education, and presence of an *APOE* ε4 allele. Finally, Aβ amyloid retention in the frontal cortex, precuneus, and anterior cingulate gyrus correlated with language (naming) performance, also prior to correction for the above co-variates. Overall, our analysis suggests that MCI and memory and naming performance depends on regional Aβ burden in non-demented PD patients.

Our study provides preliminary evidence that Aβ amyloid may not accumulate equally in all brain regions in PD patients with MCI. We found significantly higher ^18^F-florbetapir binding in the posterior cingulate gyrus of PD-MCI participants compared to PD-NC. Global measures of cognition appear to be insensitive to Aβ amyloidosis in this region once adjusted for age, sex, disease duration, education, and presence of an *APOE* ε4 allele. However, DRS-2 memory subscore inversely correlated with ^18^F-florbetapir binding in this region, even after making the same adjustments. HVLT-R immediate free total recall score showed a similar association, although after adjustment the correlation was not significant (p = 0.054). Age, education, and disease duration are known contributors to cognitive performance in PD so it is not surprising that these co-variates strongly affected our analysis. Our cohort also included one individual with a relatively lower DRS-2 memory subscore and HVLT-R score (Fig 4). This participant was not demented despite their poor performance and we did not find any technical reasons to suggest their testing results were erroneous. Nevertheless, this participant’s performance contributed significantly to our HVLT-R finding. Although outlying data points are perhaps not unexpected in small cohorts of heterogeneous and clinically complex participants, our findings should be interpreted as preliminary until validation in a larger cohort of non-demented PD patients.

The posterior cingulate gyrus is a highly interconnected region that is part of the default mode network, a robust cerebral network that is most active during periods of rest and has been reported using non-invasive methods to show abnormalities in both PD and AD [29–31]. If Aβ amyloid preferentially accrues in this region, it could serve as a biomarker for MCI with utility during relatively early stages of cognitive impairment in PD. This possibility should be tested in future longitudinal studies of cognition in PD. Additional studies are also necessary to fully characterize the functional consequence of Aβ amyloid deposition in this region in PD patients, and whether Aβ deposition here influences aggregation of α-synuclein. All other regions in PD-MCI participants also had higher Aβ amyloid retention than PD-NC participants, but these differences were not statistically significant, in part reflecting the relatively small sample size of PD-MCI patients (N = 19).

Our results suggest that composite ^18^F-florbetapir PET imaging, assessed either qualitatively by expert readers or quantitatively, does not identify PD patients with MCI. This result is perhaps not surprising since only 30% of PDD patients meet criteria for significant AD neuropathologic change at autopsy, while the remaining have alternative etiologies including cortical LB, tauopathy, or vascular injury [5, 6]. Thus, it is expected that most non-demented but cognitively impaired PD patients will have negative Aβ amyloid PET scans [11]. There have been relatively few studies of Aβ amyloid neuropathologic change in non-demented PD patients (as compared to demented PD patients), but our results suggest that in the absence of dementia, the prevalence of significant Aβ amyloid pathology is probably low. We previously examined 42 carefully followed PD patients for Aβ amyloid pathology and found that only two of 20 non-demented cases had any AD neuropathologic change [32]. In another autopsy study of 22 PD patients, four were not demented and of these, two had Aβ amyloid plaque burden of CERAD level C criteria [33]. A more recent study of 56 PD patients at autopsy quantified Aβ amyloid plaque pathology using semi-quantitative methods, and showed that Aβ amyloid plaque scores in non-demented cases were approximately one-third the scores in demented cases [34]. Importantly, ^18^F-florbetapir PET imaging was not developed to reliably detect Aβ amyloid pathology seen in subjects with low probability AD neuropathology change [35]. However, using PiB PET imaging, a previous study of non-demented PD patients also found that PiB PET imaging did not identify those with MCI at baseline [36], although those who declined cognitively over time had higher levels of cortical Aβ amyloid. It remains to be determined if baseline ^18^F-florbetapir PET imaging in non-demented PD patients can predict future conversion to dementia.

There are additional possible explanations for why composite Aβ amyloid imaging did not correlate with a diagnosis of MCI. First, we excluded participants with dementia in our study because we were specifically interested in the effect of Aβ amyloidosis on relatively early cognitive decline in PD patients. However, significant Aβ amyloid PET retention might be common only in dementia with Lewy body (DLB) patients, who are more likely than PD patients to have Aβ neuropathology at autopsy [37]. PiB studies that combine Lewy body (LB) diseases (PD, PDD, and DLB) have found higher PiB uptake primarily in those with DLB [38, 39] or not at all [40]. A recent meta-analysis of Aβ amyloid PET imaging with PiB confirmed that DLB patients have highest PiB retention among LB diseases, followed by PDD and then PD-MCI [41].

Second, Aβ amyloid imaging might correlate better with cognitive test performance measured as a continuous variable rather than dichotomized cutoff points typically applied to adhere to diagnostic criteria [18]. Mean neocortical PiB distribution volume ratio in PD patients correlated with worse performance in a composite of neuropsychological tests [42], and here we found significant inverse correlations between specific cognitive test performance and regional SUVr values. However, the useful dynamic range of our neuropsychological tests may have been restricted because demented participants were excluded.

The PIGD motor subtype has been associated with positive PiB PET imaging, which suggests that cortical dysfunction could contribute to features of advanced motor disease in PD [20, 43]. We were not able to replicate this finding using ^18^F-florbetapir PET, possibly because of the slightly different method we used to ascertain PIGD status [19]. Larger prospective studies are likely needed to determine the relevance of abnormal Aβ amyloid PET imaging to loss of postural stability and the PIGD motor subtype.

We used a probabilistic atlas method to calculate volume-weighted ^18^F-florbetapir SUVr values in segmented brain regions [21]. Importantly, this automated quantitative method had good agreement with the qualitative interpretation of the scans by expert radiologists, as previously shown in a smaller cohort [14]. An SUVr cutoff value of 1.10 was able to separate negative ^18^F-florbetapir scans from positive scans in PD patients, as it does in AD [21, 25, 26], although we did not test different cutoff points. The probabilistic atlas quantification method used in this study is an established quantification strategy for this radiotracer, although our study is one of the first applications in PD patients. This method was implemented due to lack of suitable MRI for tissue segmentation. In AD, a comparison of the probabilistic method to MR segmentation showed a similar ability to discriminate disease from controls [44]. However, the probabilistic atlas quantification method is more likely to underestimate relationships with cognition. Future studies should incorporate new ways of quantifying ^18^F-florbetapir in PD patients similar to work being done in AD [45, 46]. We also did not analyze the left and right hemispheres independently, which could be important given the asymmetry in motor PD symptoms.

The *APOE* ε4 allele is associated with cerebral Aβ amyloid deposition in older healthy controls and patients with either MCI or AD. In neuropathologically confirmed PD, the *APOE* ε4 allele is more prevalent in cases with higher burdens of coexisting Aβ amyloid pathology [47, 48], and the entorhinal, temporal, and parietal cortices could be particularly vulnerable to Aβ amyloid pathology in *APOE* ε4 carriers with PD [34]. The *APOE* ε4 allele is also more prevalent in PDD and pure DLB cases as compared to controls, even when these cases do not have sufficient Aβ amyloid pathology to reach diagnostic criteria for coexisting AD [49]. To our knowledge, our study is the first to show that in PD patients, the presence of an *APOE* ε4 allele increases quantitative ^18^F-florbetapir PET retention in addition to the effect of age. Our observation extends the previous finding of higher prevalence of *APOE* ε4 in PiB PET positive individuals from a mixed group of LBD cases [50].

In non-demented PD patients, composite Aβ amyloidosis does not distinguish individuals without cognitive impairment from those with MCI. However, regional cortical Aβ amyloid burden correlates with domain-specific cognitive performance in these patients. Our study suggests that these regions might be vulnerable to AD-related brain changes and cognitive decline prior to the onset of dementia.

## Supporting information

S1 File Demographic, clinical, and PET imaging data for all subjects. (XLSX)
